# Role of bioactive fatty acids in nonalcoholic fatty liver disease

**DOI:** 10.1186/s12937-016-0191-8

**Published:** 2016-08-02

**Authors:** Eva Juárez-Hernández, Norberto C. Chávez-Tapia, Misael Uribe, Varenka J. Barbero-Becerra

**Affiliations:** 1 Translational Research Unit, Medica Sur Clinic & Foundation, Puente de Piedra 150 Toriello Guerra, Tlalpan, ZC 14050 Mexico City, Mexico; 2 Obesity and Digestive Diseases Unit, Medica Sur Clinic & Foundation, Mexico City, Mexico

**Keywords:** Fatty acids, Liver diseases, Nutrition, Risk factors

## Abstract

Nonalcoholic fatty liver disease (NAFLD) is characterized by fat deposition in hepatocytes, and a strong association with nutritional factors. Dietary fatty acids are classified according to their biochemical properties, which confer their bioactive roles. Monounsaturated fatty acids have a dual role in various human and murine models. In contrast, polyunsaturated fatty acids exhibit antiobesity, anti steatosic and anti-inflammatory effects. The combination of these forms of fatty acids—according to dietary type, daily intake and the proportion of *n*-6 to *n*-3 fats—can compromise hepatic lipid metabolism. A chemosensory rather than a nutritional role makes bioactive fatty acids possible biomarkers for NAFLD. Bioactive fatty acids provide health benefits through modification of fatty acid composition and modulating the activity of liver cells during liver fibrosis. More and better evidence is necessary to elucidate the role of bioactive fatty acids in nutritional and clinical treatment strategies for patients with NAFLD.

## Introduction

Nonalcoholic fatty liver disease (NAFLD) is a condition characterized by deposition of fat in hepatocytes of patients with no history of excessive alcohol consumption. NAFLD causes progressive liver tissue damage from simple steatosis to nonalcoholic steatohepatitis (NASH), which involves several inflammatory processes, and might progress rapidly to develop advanced liver fibrosis, cirrhosis and hepatocellular carcinoma [1]. NAFLD is associated with obesity, type 2 diabetes, hypertension and dyslipidemia; is considered the hepatic manifestation of metabolic syndrome, and it has a strong association with nutritional factors [2].

Fats have been considered to be sources of energy and components of biological membranes, however research over recent years demonstrate a diverse array of biological activities associated with fatty acids, their derivatives and other types of lipid compounds. Bioactive fatty acids encompasses a range of structures, from simple saturated fatty acids to complex molecules derived from various biological compounds, playing important roles in energy homeostasis, cell proliferation, metabolic homeostasis and in the regulation of inflammatory processes [3, 4]. Therefore bioactive fatty acids modify lipid composition, providing a biological effect in cell signaling pathways.

Dietary fatty acids are involved in hepatic lipogenesis and might play a dual role in the pathogenesis of liver steatosis as they are involved in their development and in preventing or reversing hepatic fat accumulation. Diet fatty acid composition is an important component in NAFLD development, since 15 % of liver triacylglycerol comes from the diet [5]. Patients with NASH, claim a diet richer in fat and poorer in complex carbohydrates and protein that the general population [6].

The liver is exposed to various types of lipids (fatty acids, cholesterol and triacylglycerol’s) from the diet and visceral adipose tissues, via the hepatic portal vein, an excessive free fatty acid flux into the liver via the hepatic portal vein can cause fatty liver disease and hepatic insulin resistance [7].

The dietary fatty acids differ biochemically and they are classified by the number of carbon atoms (defining their length), the configuration of hydrogen atoms around the carbon–carbon, double or triple bonds and the positions of unsaturated bonds relative to the methyl end of the hydrocarbon chain [8, 9]. These biochemical differences, condition their biological activities.

The aim of this review was to determine the role of bioactive dietary fatty acids on the modulation of biochemical and cell activity during the development of liver fibrosis.

### Fatty acid classification

#### Short-chain fatty acids

Undigested food or dietary carbohydrates in the small intestine undergo subsequent fermentation in the colon by its microbiota and give rise to various microbial metabolites such as short-chain fatty acids (SCFAs), including acetic, propionic and butyric acid [10]. These are absorbed rapidly, mostly by nonionic diffusion, but also by active transport mediated by a sodium-coupled transporter [11]. Changes in gut’s microbiota can lead to excessive energy production with negative effects on metabolism and obesity, promoting liver steatosis [12]. In murine models, feeding a mixture of SCFAs decreased the hepatic cholesterol synthesis rate [13], while high fat consumption promotes harmful inflammatory effects that seem to be partly counteracted by SFCAs, specifically propionic and butyric acids [14]. Thus, NAFLD has been associated with a shift in the gut microbiota profile, and treatment with probiotics has been suggested to prevent the progression of this liver disease [15, 16].

#### Butyrate/butyric acid

Among SCFAs, butyrate is the primary energy source of colonocytes, it is one of the main products of fiber fermentation, as well is a poorly digestible polysaccharide in the colon and distal small intestine [17], specifically of digestion-resistant starches and dietary fiber types, but also it comes to some extent from dietary and endogenous proteins. After the absorption of butyrate by the colon, colonocytes metabolize it in part and the remaining fraction reaches the liver via the hepatic portal vein [7]. In particular, butyrate has the ability to enhance the growth of lactobacilli and bifidobacteria in the colon [11], also has various beneficial metabolic effects such as improving thermogenesis and energy expenditure, which contributes to reduce body weight and other markers of metabolic syndrome [18]. For instance, the probiotic strain *Clostridium butyricum* (MIYAIRI 588) produce butyrate in a murine model of a choline-deficient/L-amino acid defined diet, which prevents the progression of liver damage through a reduction in hepatic lipid deposition and improve triacylglycerol content and insulin resistance [15]. Sodium butyrate and a synthetic butyrate derivative showed beneficial effects on subjects with NAFLD, preventing liver inflammation, metabolic impairment and reducing insulin resistance [15]. Moreover, butyrate induced the production of fibroblast growth factor 21, which is involved in stimulating hepatic fatty acid β-oxidation [19].

#### Propionate/propionic acid

Propionic acid (PA) is produced naturally in a few food products, such as milk in small amounts, but in higher levels on dairy products like yogurt and cheese following bacterial fermentation, mainly by propionibacteria [20]. Undigested carbohydrates, such as dietary fiber and digestion-resistant starch represent the major sources of PA [20]. The quantity of PA produced depends on the microbiota, the type and quantity of the substrates and gut transit time [21]. The liver metabolizes around 90 % of PA and the rest is transported into the peripheral blood system [10].

Propionate is involved in hepatic cholesterol synthesis rate [13] and in high-density lipoprotein and triacylglycerol levels regulation [22]. It has been considered a substrate for hepatic gluconeogenesis, but it seems to have two competing and contrasting effects, regards of being a substrate or inhibitor of gluconeogenesis [21]. Moreover, a role in other disorders such as poor control of satiety has been studied [23]. Clearly, a well-designed study to test the efficacy of SCFAs in treating liver diseases is imperative.

#### Acetate/acetic acid

Acetic acid is frequently ingested in the diet given that vinegar is a common condiment; however oral commensal bacteria are another source of acetate [24]. Acetate is considered the most abundant SCFA in the foregut lumen and the principal in the colon. It is absorbed immediately after ingestion and is transported directly to the liver, so is less metabolized in the colon [21]. Even though it is believed to be similar to propionate on having a low-level role in cholesterol metabolism, it seems that acetate is more effective in this regard. [25] Moreover, acetate in the intestinal lumen activates multiple mucosal responses, including the release of gut hormones and afferent nerve activation [26].

#### Medium-chain fatty acids

Medium-chain fatty acids (MCFAs), with 8–10 carbon atoms, are found as triacylglycerols in many foods. They are found in palm kernels and coconut oil, butter, fresh cream and milk, which are considered the main sources of MCFA [9]. Medium-chain triacylglycerols (MCTs) have rapid absorption and high solubility, which precludes their rapid hydroxylation to MCFAs. They are transported directly into the liver via the hepatic portal vein, here they are metabolized rapidly by β-oxidation, increasing diet-induced thermogenesis [27]. There is evidence that MCFAs are essentially oxidized to acetyl-coenzyme A within hepatic mitochondria and further degraded to CO_2_ or converted to ketone bodies [28]. Dietary MCTs induce thermogenesis and do not contribute to weight gain because they are not deposited in adipose tissues; therefore, MCTs might be used as a medium of preventing and treating obesity [18].

**Fig. 1:**
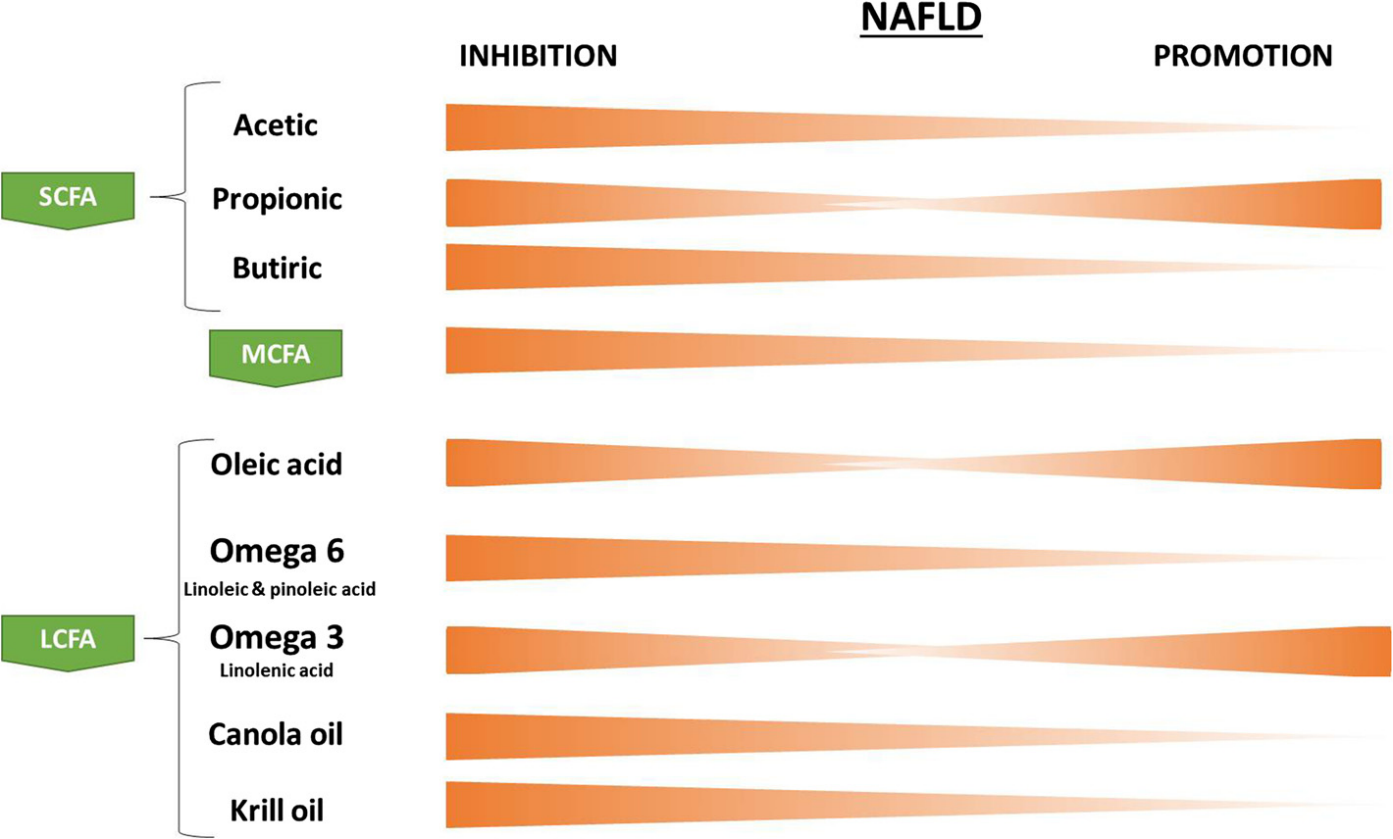
Bioactive fatty acid patterns according to nonalcoholic fatty liver disease (NAFLD) inhibtion and promotion. *Key*: The color bars tapering toward either side of the outcome line indicate a decline in activity of the NAFLD stage (orange) according to each fatty acid. The bioactive fatty acids panel includes SCFAs, MCFAs, and LCFAs. *Key*: NAFLD, nonalcoholic liver disease; SCFA, short-chain fatty acid; MCFA, medium-chain fatty acid; LCFA, long-chain fatty acid

After consumption of a MCT-rich oil diet in mice, plasma triacylglycerol levels were elevated, as were hepatic lipogenic enzymes activities, acetyl-coenzyme A carboxylase, fatty acid synthase and diacylglycerol acyltransferase; however the levels of plasma and hepatic cholesterol were reduced [28]. A MCFA-rich diet with replacement of dietary long-chain triacylglycerols has been shown to reduce the levels of steatosis and the markers of hepatic injury, such as hepatic transaminases. This replacement appeared to result in a substantial upregulation of fatty acid liver oxidation, sufficient to reduce the development of steatosis [29]. The substitution of saturated for unsaturated fats provides a dose-responsive protective effect on the development of alcoholic liver disease in a murine model of ethanol consumption in vivo [30]. The severity of steatosis varies widely depending on the dietary saturated fat content, so delineation of the type of dietary fat is an important factor that affects hepatic pathology by inducing or promoting the development and progression of NAFLD (Fig. 1).

In a study performed in slightly obese subjects with a body mass index ≥25 kg/m^2^, the body weight loss was higher in the group that was eating bread (containing 14 g of oil as MCTs daily) for breakfast, than in the group ingesting long-chain triacylglycerols (LCTs) [31]. As MCTs increase energy expenditure, this could result in a faster feeling of satiety and facilitate weight control, when included in the diet as a substitute for fats containing LCTs [32]. MCTs have certain disadvantages when cooked, that it has limited their general use [9], such as a low smoking point and excessive foaming when used for deep frying, a balance between MCTs and LCTs could help in suppressing body fat accumulation.

MCFAs have been employed in certain clinical tests. For example, C-octanoic acid has been used as a substrate in noninvasive breath testing to assess total body β-oxidation in subjects with NASH [33]. In animal models, the therapeutic supplementation of caprylic acid could effectively decrease the levels of *Campylobacter* species of pathogenic bacteria [34].

#### Long-chain fatty acids

Long-chain fatty acids (LCFAs) are molecules containing 12 or more linearly arranged carbon atoms, which are found mostly as components of the triacylglycerols of edible oils and fats [18]. Long-chain triacylglycerols are hydrolyzed at the 1,3-ester bond of glycerol by pancreatic lipase in the small intestine, mainly to 2-monoacylglycerol. This product together with other fatty acids are dissolved in micelles and absorbed by mucosal cells in the small intestine. LCFAs are re-synthesized to triacylglycerol, form chylomicrons and then are released into the circulation via the lacteal lymph vessels and transported to peripheral tissues [9].

Fatty acid composition is characterized by the degree of unsaturation in the fatty acid chain and the molecular configuration at the bonds [8], where fatty acids may be saturated (no double bonds) or unsaturated (one or more double bonds). These features produce specific biological functions.

#### Monounsaturated fatty acids

Oleic acid is a monounsaturated fatty acid that naturally occur in various animal and vegetable fats and oils. Previous studies have shown a dual role of oleic acid. Thus, in murine and human hepatocyte models, treatment with palmitic acid triggered the early activation of stress-related kinases and apoptosis, which were attenuated by oleic acid treatment [35]. Oleic acid was able to induce liver X-receptor alpha mRNA synthesis in human neutrophils, but it led to decreased intracellular lipid levels and inflammation markers [36]. Conversely, in an in vitro model, oleic acid induced hepatic steatosis through aquaporin 3 and 9 via p38-signaling regulation [37]. In an in vitro model of steatosis, induced by oleic acid, treatment with phenolic acids led to increased fatty acid oxidation and the synthesis of triacylglycerol and cholesterol [38]. Moreover, high plasma levels of oleic acid were observed in a clinical study including 10 patients with NAFLD and 9 with NASH [39]. Further studies are needed to understand the cellular and physiological processes involving oleic acid and elucidate its role in NAFLD.

#### Polyunsaturated fatty acids

Polyunsaturated fatty acids (PUFAs) include linoleic acid (*n*-6), alpha-linolenic acid and arachidonic acid. The simplest *n*-6 fatty acid is linoleic acid (C18:2), while linolenic acid (C18:3) is the simplest *n*-3 fatty acid. Murine models have demonstrated effects against obesity [40], steatosis and infflamation from *n*-3 fatty acids [41]. In addition, dietary supplementation with PUFAs combined with hypocaloric diets had a beneficial effect on plasma lipid profiles [42].

Several types of oils containing PUFAs have shown to prevent the onset of NAFLD. Canola oil, which is produced from the seeds of several varieties of plants, contains linoleic acid (*n*-6) and α-linolenic acid (*n*-3) in a 2:1 ratio. Hanke et al. observed that hepatic steatosis was attenuated in mice treated with different variants of canola oil; however, increased levels of eicosapentaenoic acid (EPA) and docosahexaenoic acid (DHA) were not correlated with markers of inflammation or with mRNA levels encoding for desaturated or elongated PUFAs [43].

Krill oil, which also contains *n*-3 PUFAs, EPA and DHA, has beneficial effects on glucose and lipid metabolism, as well on the mitochondrial respiratory chain [44]. These effects could be explained by the stimulation and catabolism of excess fats introduced by a hypercaloric diet, while the fatty acid synthesis de novo is inhibited [5]. Moreover, the combination of DHA and EPA with alpha-linolenic acid can lower the risk of fatal ischemic episodes [45].

However, the role of PUFAs in diet is still controversial and their effects on inflammation and fibrosis during steatohepatitis remain unresolved. In a steatohepatitis murine model fed with a methionine-choline-deficient (MCD) diet supplemented with olive oil (OO) and/or *n*-3 PUFAs, higher alanine transaminase levels where induced and more severe scores of inflammation, including a significant increase in lipogranulomas number and intrahepatic gene expression of proinflammatory and profibrogenic molecules, that when the diet was supplemented only with OO [46].

Recently it has been observed that some oxidized lipids from PUFA’s, play an important role on inflammatory pathways, therefore, these could be associated to the development and progression of NAFLD, however more studies are needed to establish the association with oxidized lipids and the progression of NAFLD to NASH or HCC [47].

The ‘daily recommended intake’ is a very important concept to consider; a cross-sectional study evaluated the dietary intake in patients with NAFLD, showing that more than 80 % of them did not reach the daily recommended intake of linolenic and linoleic acids [48]. However, the intake of PUFAs was higher in NAFLD patients, suggesting that this factor alone might not be a determinant for NAFLD. Thus, new bioactive molecules need to be discovered in this context [48].

#### n-6 versus n-3 fatty acids

Because dietary *n*-3 PUFAs are able to limit triacylglycerol storage in the liver, it is necessary to consider the *n*-3/*n*-6 ratio, given that high *n*-6 levels could induce NAFLD and other chronic diseases [5]. A higher *n*-3/*n*-6 ratio in the diet is desirable as a way of improving human health by reducing the weight of intra-abdominal fat, reducing adipocyte size and normalization of the heartbeat. This is because *n*-3 PUFAs are usually converted to anti-inflammatory eicosanoids while *n*-6 PUFAs are converted to proinflammatory eicosanoids [49].

The n-3/n-6 ratio has an important association with the development of NAFLD, where a diet with a higher *n*-6/*n*-3 ratio and suboptimal PUFA intake could result in a hepatic lipid metabolism dysregulation, insulin homeostasis and inflammatory pathways disarrangement [48]. Patients with NAFLD showed a significant decrease in the *n*-6/*n*-3 ratio, the same scenario was observed in patients with NASH compared with healthy controls [39]. Although patients with NASH had a lower intake of either *n*-3 or *n*-6 fatty acids than controls, such patients had a significantly higher intake of *n*-6 fatty acids and a decreased *n*-6/*n*-3 ratio versus controls have been reported, which have been shown to have a negative impact on health maintenance and disease prevention [6]. The increased *n*-6/*n*-3 ratios in patients with liver diseases have different causes, such as inadequate intake of precursors and a higher intake of *n*-9 *trans* isomers [39].

It is clear that is necessary to maintain an adequate *n*-6/*n*-3 ratio to avoid the development of insulin resistance and the activation of inflammatory pathways involved with fat deposition in the liver. Allard et al. suggested that *n*-3 dietary supplementation in addition to lifestyle modifications might be beneficial in preventing the progression of steatosis and ensures an adequate balance between these fatty acids [50]. Despite the findings about the effect of PUFA’s on NAFLD prevention, it is not clear if an adequate diet including PUFA’s could reverse the progression stages of NASH or liver fibrosis [47].

With respect to *n*-6 PUFAs, pinolenic acid is characterized by having polymethylene-interrupted double bonds, it is contained in pine seed oil, exhibiting beneficial effects on lipid metabolism [51]. In a murine model with a diet that contains pine seed oil, a significant increase in the splenic production of immunoglobulins and leukotrienes was shown [52]. Moreover, in a murine model, pinolenic acid exerted antidiabetic effects as well as some beneficial metabolic effects such as reducing weight gain and intramuscular lipid accumulation [53]. These findings suggest that pine seed oil might have potential effects as a dietary supplement to counteract obesity and metabolic dysregulation [54].

### Bioactive fatty acids as biomarkers

Based on studies suggesting that bioactive dietary fatty acids can suppress the accumulation of abdominal adipose tissue and serum lipids, the primary function of bioactive fatty acids absorbed by the duodenum might be chemosensory rather than nutritional [26]. Thus, the proposal of using bioactive fatty acids as biomarkers in patients with NAFLD could be attractive. Glucagon-like peptide 1 (GLP1) is involved in the development of NASH, administration of a peptide agonist of the GLP1 receptor (Exendin-4) improved steatohepatitis through the increase of hepatic long chain saturated fatty acid levels and a hepatic *n*-3/*n*-6 PUFA ratio reduction, specifically by the regulation of hepatic fatty acid metabolism [55]. Furthermore, high levels of acid sphingomyelinase are present in the serum of patients with NAFLD [55, 56]. While treatment with a cholesteryl ester transfer protein reduced HDL levels, it might also increase the risk of atherosclerosis [57].

There are important roles for the phospholipase A family (PLA) in several diseases. Phospholipase A2 (PLA2) levels were significantly increased in patients after ischemic liver tissue versus nonischemic controls [58]. Moreover, PLAs have distinct roles in diet-induced obesity, such as counteracting adipose tissue inflammation, insulin resistance, hyperlipidemia and obesity through facilitating lipid accumulation in adipose tissue [59]. Another phospholipase member, calcium-independent phospholipase A2 has been shown to act as an upstream checkpoint for mechanisms that regulate fatty acid uptake [60].

### Mechanisms involving receptor recognition

The nutrient receptors include all known G protein-coupled receptors (GPCRs) or free fatty acid receptors, which are a large class of seven transmembrane proteins that regulate a diverse range of signaling events and are important in mediating autocrine, paracrine and endocrine functions [61]. These have specificity according to free fatty acid or lipid molecule type binding and activation status.

#### Short-chain fatty acids

Within the GPCR gene superfamily GRP40–43 is a phylogenetically related group which comprises tandemly encoded genes and shares approximately 30 % minimum identity [62]. According to the SCFA type, acetate and propionate produce a reduction in lipolytic activity through GPR43 activation in mice studies [63]. However, an increase of lipids in adipocytes via peroxisome proliferator-activated receptor gamma 2 upregulation has also been observed [64]. Acetate is an agonist for GPR43 and GPR41 receptors; in humans, GRP43 has a higher affinity to acetate than does GPR41, but in mice, GPR43 and GPR41 are activated equally by acetate [65]. Butyrate has multiple effects, which involve several distinct mechanisms of action such as epigenetic gene regulation [66], acting as a signal molecule, targeting GPR43, GPR41 [62] and GPR109A [67]. A study using sodium butyrate demonstrated a reduction in proinflammatory cytokine and chemokine levels in a steatosis rat model, via toll-like receptor and nuclear factor kappa-B inhibition, as well PPAR-α recovery with the probable involvement of peroxisome proliferator-activated receptor gamma and coactivator 1 alpha (PGC-1α) [7]. Moreover, butyrate improves lipid profiles by enhancing tight junction protein expression, and by decreasing the levels of ALT, TNF-α and serum endotoxin, apparently through 5′adenosine monophosphate-activated protein kinase-related, nuclear factor erythroid-derived 2, sterol regulatory element binding protein, PPARγ and NHE8 (sodium/hydrogen exchanger 8) expression [15], and the enhanced Sp3 (Sodium butyrate-mediated acetylation) interaction [68].

Evidence suggests that cell-surface receptors contribute to extracellular actions of SCFAs; however, intracellular actions also play an essential role in the biological effects and involve their function as potent epigenetic modifiers related to the ability of butyrate and propionate to inhibit histone deacetylases (HDACs), but not acetate. To successfully achieve this intracellular function, SCFAs enter the cell through the Na+-coupled high-affinity Slc5a8 (solute carrier gene family 5a, member 8) transporter in order to have access to HDACs [67, 69]. Furthermore, butyrate promotes immune cell conversion that consequently blockade dendritic cell development; however, this mechanism has been related with dietary fiber content conditions, where transporter would become essential only when dietary fiber intake is sub-optimal [69].

According to lipid modifications, S-acylation is a unique dynamic process that is reversible and promotes the regulation of the subcellular distribution of key signalling molecules, which explains the importance of protein localization at several sites in the cell and hence their function [70]. At mitochondrial level, dietary SCFAs act through the repression of PPARγ expression, subsequently AMP/ATP ratio increase leading to the activation of AMPK and culminating in improving oxidative metabolism in the liver and adipose tissue [71].

#### Medium-chain fatty acids and long-chain fatty acids

MCTs, precursors of MCFAs, are thought to enter hepatocytes and mitochondria by a minimal use of the normal fatty acid transport systems or by diffusion, and then degraded by direct thermogenesis [72]. MCFAs are not incorporated into chylomicrons, instead they are absorbed directly into the hepatic portal vein, suggesting that they do not require transporters such as CD36 or fatty acid transporter proteins to enter the liver [29, 72]. However, it has been demonstrated that GPR40 and GPR120 receptors are involved with MCFA and LCFA uptake respectively. These receptors are expressed in enteroendocrine L and K cells producing glucagon-like peptides (GLP-1 and 2) and gastric inhibitory peptides [73–75]. MCFAs have several recognition and activation mechanisms that need to be elucidated.

LCFAs (*n*-3) are involved in liver steatosis and inflammation through decreased PPAR-α signaling and NF-kB activation [41]. However, feeding mice with a HFD aggravate liver injury increasing the expression levels of proinflammatory and oxidative stress molecules, as well as PUFA-oxidizing enzymes in a murine lipopolysaccharide model [76]. Regarding other GPCR members, GPR119 has been involved in decreasing cellular cholesterol contents and inflammation inhibition, apparently through the GLP-1 receptor signaling pathway in an in vitro mice cell model [77]. GPR120 is highly expressed in the digestive system, adipose tissues, lung, macrophages and also is present in the endocrine pancreas; it seem to be specific for long-chain unsaturated fatty acids, including *n*-3 PUFAs [78] where it appears to be dispensable for the improved metabolic profile associated with diets enriched with these PUFAs [79].

In autoimmunity diseases, MCFAs or LCFAs, such as lauric acid or palmitic acid, promote differentiation of naive T cells into T helper 1 (Th1) and Th17 cells, promoting inflammation apparently through activation of the p38 MAPK pathway. On the other hand, SCFAs, propionate most potently, enhanced polarization of T cells toward Treg cells probably through lipin2-JIP2 pathway, promoting suppression of inflammation. Triggering factors have not been identified yet; however, cell-surface receptors are the most likely targets [80, 81].

Moreover, several studies have been described as key molecules that seem to be related to fatty acid regulation mechanisms. A microsomal enzyme named after Evolv- 6 has been involved in saturated and monounsaturated fatty acids regulation and in modifying fatty acid composition. In a LDL receptor-deficient murine model, absence of Evolv-6 showed a double effect in metabolism of fatty acids with a decrease in lipid accumulation, cholesterol levels, hepatic inflammation and oxidative stress, which leads a direct effect in NAFLD progression [82]. Sterol regulatory element-binding proteins (SREBPs) are transcription factors that activate the synthesis of FAs, triglycerides (TGs), and cholesterol; SREBPs overactivation in liver cause TG accumulation and hepatic steatosis. Thus, in a murine model it has been showed that the elimination of nuclear SREBPs, prevents hypertriglyceridemia induced by carbohydrate accumulation [83]. On the other hand, a membrane bound protein (SREBP-2), activates enzyme-cholesterol biosynthesis genes, exerting an important role on cholesterol homeostasis [84].

Although the published evidence shows discrepancies, we should mention that the beneficial health effects of PUFAs could be dependent on their *cis*-isomer configuration, which is the predominant bioactive form. [85] Fatty acids in their configuration have a rigid nonlinear structure, which enhances membrane fluidity when incorporated into cells, promote cell-to-cell communication and help to maintain normal homeostasis or prevent the development of metabolic disorders [18]. The benefits of PUFAs could be associated with their functions in mediating transcription factors involving the expression of genes inplicated in lipid synthesis and oxidation [42, 86]. Thus, biochemical configuration and gene expressions should be considered to explain the observed discrepancies in the actions of PUFAs.

## Discussion

Fatty acid composition and carbon chain length are both important in terms of their impact on human health. The fat type in the diet affects the rate of hepatic triacylglycerol synthesis, which is an important determinant of plasma triacylglycerol concentrations [28]. Moreover, the bioavailability of fatty acids is another parameter to consider [42]. Alcohol ingestion along with PUFAs aggravates the production of free radicals and aggravate oxidative stress [87]. Consumption of foods with bioactive components within an energy-restricted diet could be an option for the treatment of patients with NAFLD. However, the quality and combination of macronutrients are more important than their isolated amounts.

## Conclusion

**Fig. 2:**
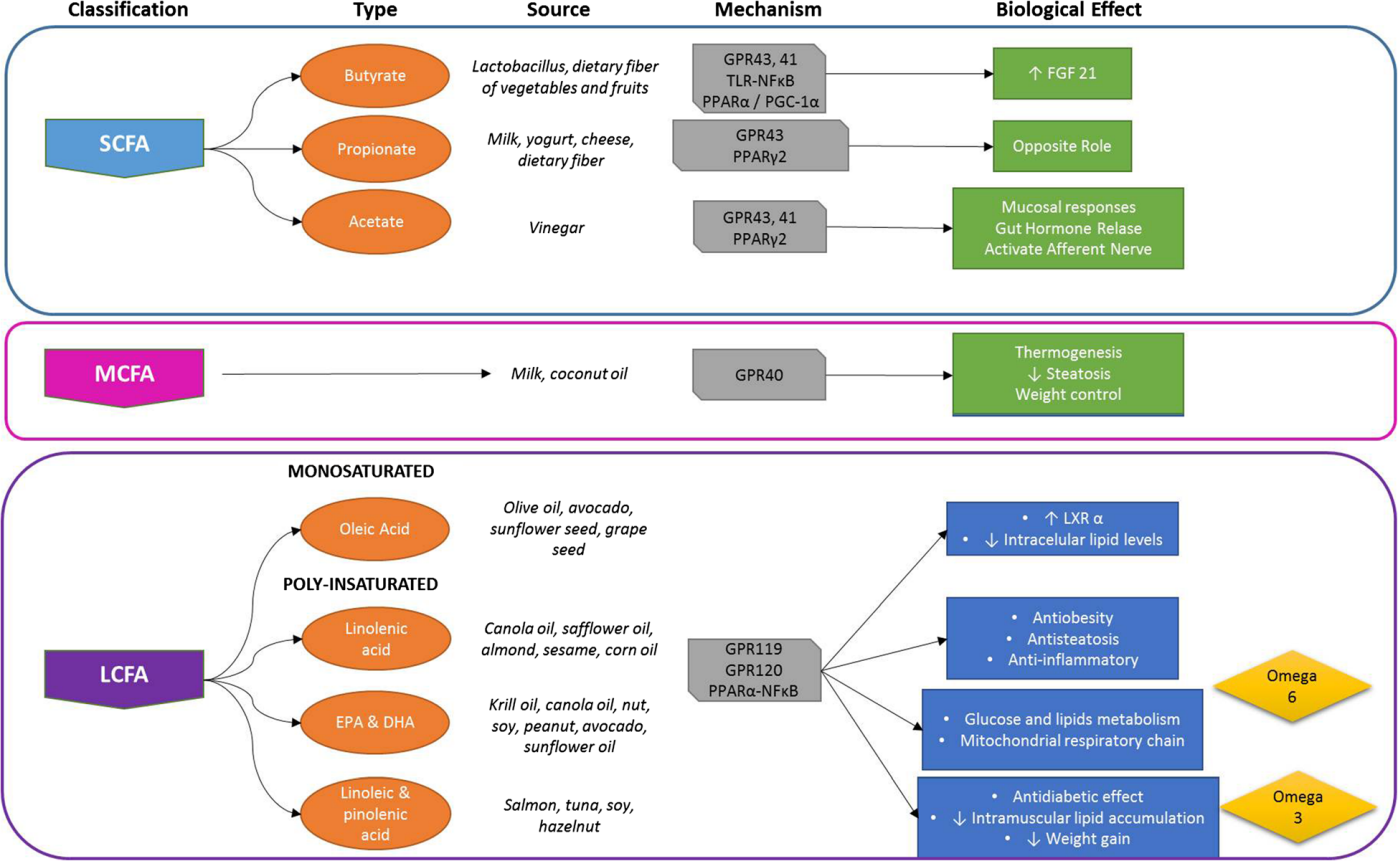
Bioactive fatty acids function in the development of nonalcoholic fatty liver disease. *Key*: describing the different types of bioactive fatty acids in relation to their sources, cell recognition mechanisms and biological effects

**Fig. 3:**
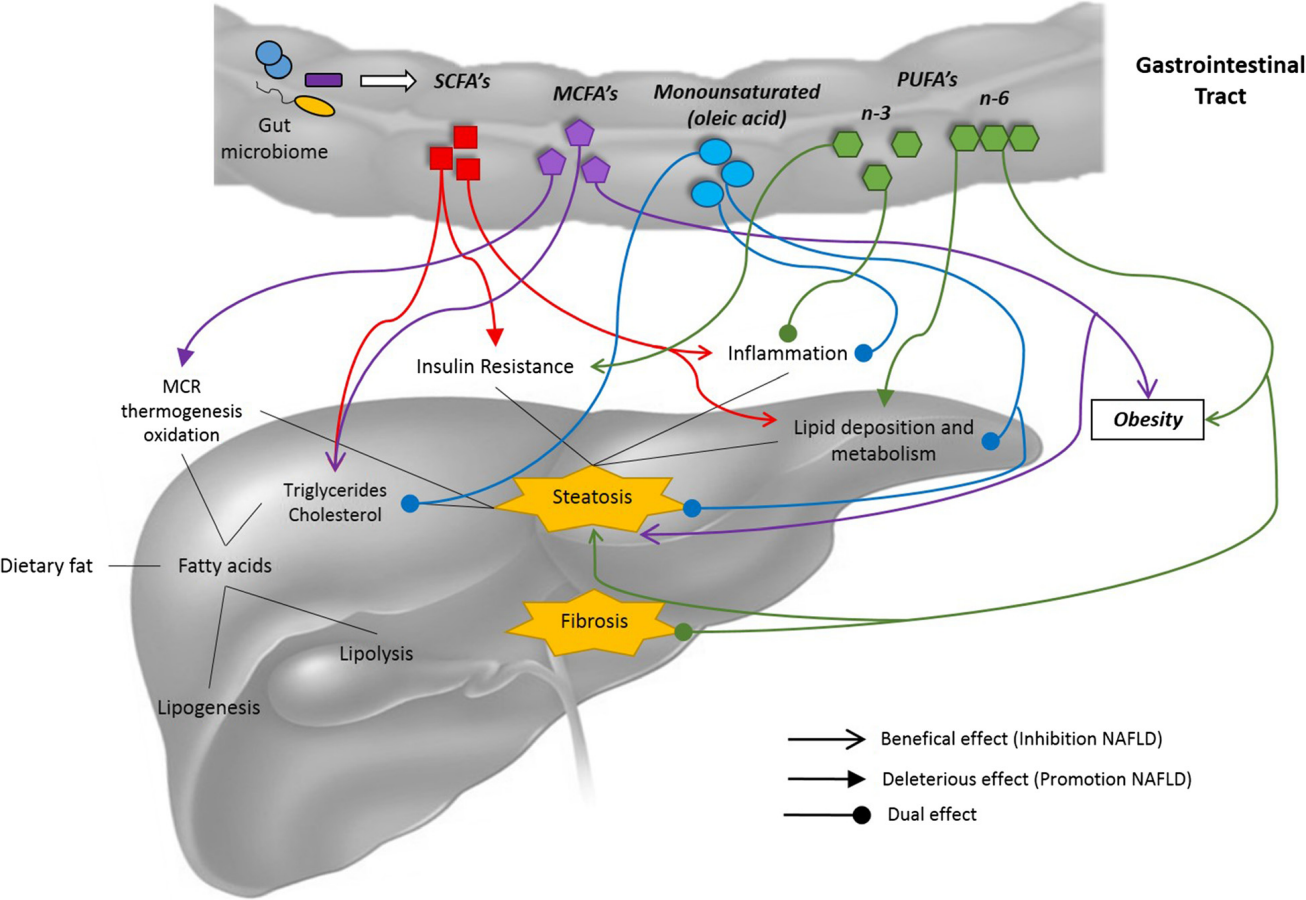
Interaction between bioactive fatty acids and NAFLD development. *Key*: MCR, mitochondrial respiratory chain; SCFAs, short-chain fatty acid; MCFAs, medium-chain fatty acid; PUFAs, polyunsaturated fatty acid

The idea that several groups of lipids might provide health benefits through changes in the tissue fatty acid compositions or the induction of cell signaling pathways is intriguing (Fig. 2). It seems that bioactive fatty acids are involved in modulating the activity of all cell types of the liver during the development of obesity, NAFLD and liver fibrosis (Fig. 3). However, more and better evidence is required regarding the roles of bioactive fatty acids in humans, given the complicated clinical nature of NAFLD. Improved methodological designs and larger sample sizes are necessary to make decisions about whether to include bioactive fatty acids in nutritional and clinical treatment strategies for patients with NAFLD

## Abbreviations

NAFLD, nonalcoholic fatty liver disease; NASH, nonalcoholic steatohepatitis; SCFAs, short-chain fatty acids; PA, propionic acid; MCFAs, medium-chain fatty acids; MCTs, medium-chain triacylglycerols; LCTs, long-chain triacylglycerols; LCFAs, long-chain fatty acids; PUFAs, polyunsaturated fatty acids; EPA, eicosapentanoic acid; DHA, docosahexanoic acid; MCD, methionine-choline-deficient; OO, olive oil; GLP1, glucagon-like peptide 1; PLA, phospholipase A family; GPCRs, G protein-coupled receptors; PGC-1α, peroxisome proliferator-activated receptor gamma and coactivator 1 alpha; HDACs, histone deacetylases; SREBPs, sterol regulatory element-binding proteins; TGs, triglycerides
